# Efficacy and Safety of Remote Cardiac Rehabilitation in the Recovery Phase of Cardiovascular Diseases: Protocol for a Multicenter, Nonrandomized, Single-Arm, Interventional Trial

**DOI:** 10.2196/30725

**Published:** 2021-10-04

**Authors:** Hidetaka Itoh, Eisuke Amiya, Koichi Narita, Mai Shimbo, Masanobu Taya, Issei Komuro, Takashi Hasegawa, Shigeru Makita, Yutaka Kimura

**Affiliations:** 1 The University of Tokyo Hospital Tokyo Japan; 2 Japan Telemedicine Society Gunma Japan; 3 Saitama Medical University International Medical Center Saitama Japan; 4 Kansai Medical University Osaka Japan

**Keywords:** cardiac rehabilitation, remote system, e-learning, exercise capacity, rehabilitation, cardiovascular disease, monitoring system, disease prevention, cardiology

## Abstract

**Background:**

Conventional group-based outpatient cardiac rehabilitation through monitoring and center-based approaches for patients in the recovery phase has shown strong evidence for the prevention of cardiovascular diseases. However, there are some cases in which maintaining attendance of center-based cardiac rehabilitation is difficult.

**Objective:**

This study aims to ascertain the safety and efficacy of remote cardiac rehabilitation (RCR) in the recovery phase in patients with cardiovascular disease.

**Methods:**

Patients satisfying the study criteria will be recruited from multiple institutions (approximately 30) across Japan. In total, 75 patients (approximately 2 or 3 patients from each institution) are proposed to be recruited. Patients enrolled in the RCR group will be lent devices necessary for RCR (including calibrated ergometers and tablets). Patients will perform anaerobic exercise at home using ergometer for 30-40 minutes at least 3 times weekly. During exercise, an instructor will monitor the patient in real time (using interactive video tools and monitoring tools for various vital data). Moreover, educational instructions will be given 3 times weekly using e-learning methods.

**Results:**

The primary endpoint is the peak oxygen uptake 2-3 months from the start of exercise or 6-min walk test. The extracted data will be compared between RCR patients and controls without RCR.

**Conclusions:**

The establishment of the system of RCR proposed in this study will lead to the development of more extensive applications, which have been insufficient through conventional interventions.

**Trial Registration:**

University Hospital Medical Information Network—Clinical Trials Registry UMIN–CTR UMIN000042942; https://upload.umin.ac.jp/cgi-open-bin/ctr_e/ctr_view.cgi?recptno=R000048983

**International Registered Report Identifier (IRRID):**

DERR1-10.2196/30725

## Introduction

Conventional group-based outpatient cardiac rehabilitation through monitoring and center-based approaches for patients in the recovery phase has shown strong evidence for the prevention of cardiovascular diseases [1-5]. However, maintaining attendance during center-based cardiac rehabilitation is difficult with certain patients because of the distance from their home to rehabilitation sites [5-8]. Poor program adherence is a major issue because the benefits of center-based rehabilitation depend on exercise frequency to a certain extent [9]. Moreover, because of the spread of COVID-19, this form of medical care is expected to present cluster infection–related risks. Thus, nationwide restrictions have been placed on outpatient cardiac rehabilitation (OCR) for patients with cardiovascular diseases in the recovery and maintenance phases [10-12]. Consequently, treatment for patients with high-risk cardiovascular diseases has become insufficient [13]. The suspension of OCR is expected to increase the instances of rehospitalization of patients with acute myocardial infarction and heart failure. Thus, developing alternative modalities to OCR is an urgent challenge. In recent years, reports on the possibility of remote cardiac rehabilitation (RCR) have been filed sporadically overseas [13-15]. RCR consists of health care delivery similar to that of OCR, which corresponds to monitoring during exercise, education, nutritional counseling, and psychological support via telephone and digital platforms. These include the use of artificial intelligence (AI), the Internet of Things, and e-learning technologies in monitoring the vital signs of patients staying at home, simultaneously ensuring levels of safety comparable with those of in-person monitoring by specialists [16,17]. However, since only a few reports have been available, conclusive data on the benefits of RCR are lacking. This study aims to ascertain the safety and efficacy of RCR in the recovery phase among patients with cardiovascular disease. The establishment of the system proposed in this paper will not only help patients transition from OCR to home-based care but also lead to the development of more extensive applications. In fact, the results of this study might provide some suggestions to consider more efficient ways in continued rehabilitation and disease control in the maintenance stage, which have been insufficient with conventional interventions.

## Methods

### Selection of Patients for the Study

**Figure 1:**
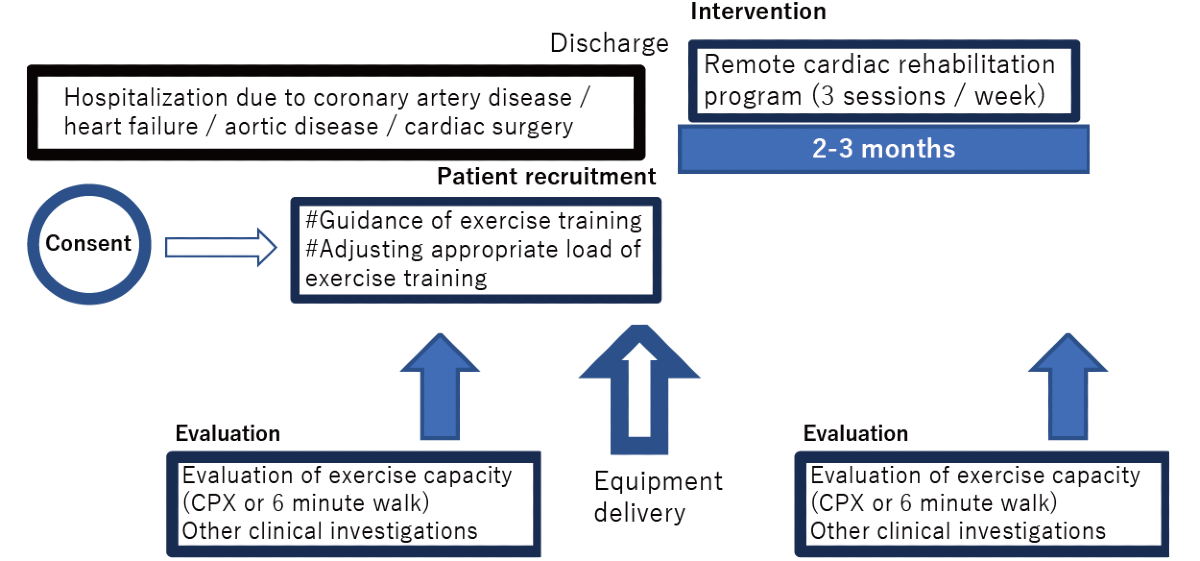
Schematic representation of the flow of remote cardiac rehabilitation in this study. CPX: Cardiopulmonary exercise test.

Patients satisfying the following criteria will be recruited from multiple institutions (approximately 30) certified by the Japanese Association of Cardiac Rehabilitation across Japan: (1) patients who are recommended by the attending physician to continue postdischarge cardiac rehabilitation following in-hospital treatment for diseases indicated for cardiac rehabilitation (including ischemic cardiac disease, heart failure, aortic disease, postcardiac surgeries, and peripheral arterial disease) and (2) patients who voluntarily consent to participate in this study with a complete understanding of thorough explanations provided. Conversely, the exclusion criteria are as follows: (1) patients aged under 20 years; (2) those who are deemed unsuitable to participate in this study by the attending physician; (3) those with complications contraindicated for exercise or with high exercise-induced risks (including highly advanced valve stenosis, serious heart failure equivalent to New York Heart Association [NYHA] classification IV, at risk of serious arrhythmia [including ventricular tachycardia], and serious renal/hepatic disease); (4) those with an implanted defibrillator or ventricular assist device; (5) those with reduced cognitive function; (6) those at a terminal disease stage; (7) those at term pregnancy; and (8) those who are determined by a researcher at each institution to be unable to safely undergo RCR (eg, patients living alone). In total, 75 patients (approximately 2 or 3 patients from each institution) are proposed to be recruited. Patient recruitment started in January 2021 and proceeded through March 2021. The study timeline is described in Figure 1.

### RCR Protocol

This study aims to enroll approximately 75 patients introduced to RCR and compare them with patients who received OCR for the same duration as that for a historical control group. The criteria for the control group are as follows: (1) patients who are recommended by the attending physician that they continue postdischarge rehabilitation following in-hospital treatment for diseases indicated for cardiac rehabilitation and (2) those who took cardiopulmonary exercise testing or a 6-min walk test at 2-3 months after discharge.

**Figure 2:**
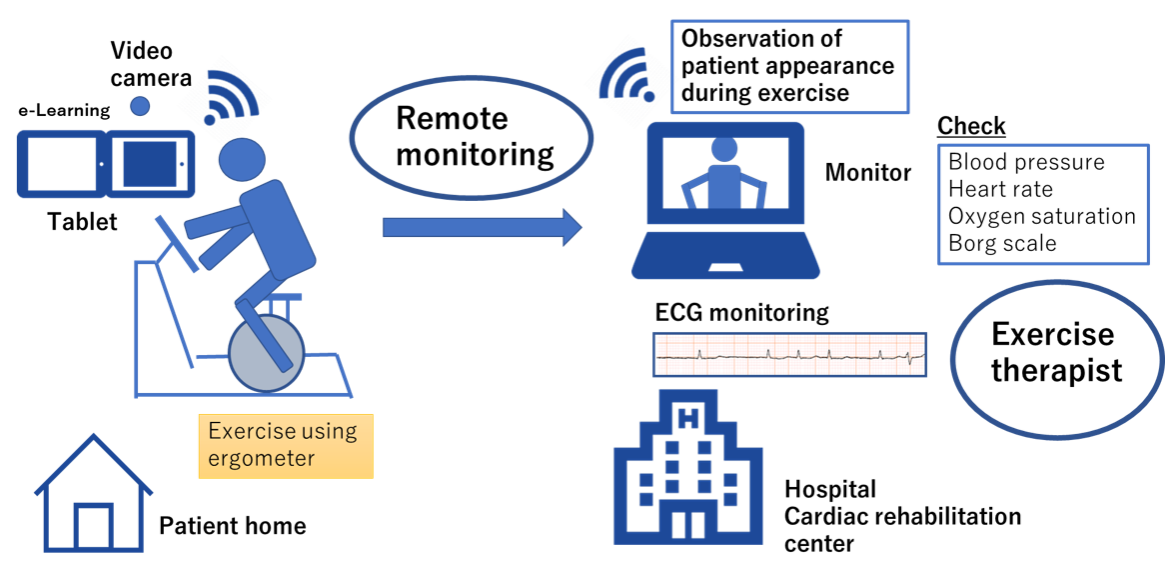
Schematic representation of remote cardiac rehabilitation in this study. ECG: electrocardiogram.

Patients enrolled in the RCR group will be lent devices necessary for RCR (including calibrated ergometers and tablets) (Figure 2). The main part of this RCR program will be anaerobic exercise using the lent ergometer. The appropriate exercise intensity in RCR will be determined during hospitalization or after discharge. The intensity levels will be set individually at the anaerobic threshold (AT), for instance, based on the heart rate at the AT, in accordance with the results of cardiopulmonary function testing [18,19]. Alternatively, intensity levels will be determined in reference to the exercise load given during hospitalization. In principle, the duration of exercise will be 30-40 min, starting from ~10 min and then gradually extended. A Borg scale score of 11-13 is the intended target exertion level. The frequency of exercise will be at least 3 times weekly. Upon initial checkup of each exercise session, body temperature, weight, blood pressure, and heart rate are recorded. During exercise, an instructor will monitor the patient in real time (using interactive video tools and monitoring tools for various vital data) and check blood pressure, heart rate, oxygen saturation, and respiratory rate regularly. Data of the electrocardiographic waveform are sent from patients to monitor located at the cardiac rehabilitation center, which could be observed by the instructor. When there are signs or symptoms that suggest that continuing exercise has some risks for worsening a patient’s state, the instructor instructs the cessation of that exercise session and, if necessary, the instructor instructs outpatient consultation. At the final checkup, the Borg rating of the perceived exertion scale is checked in addition to data on blood pressure and heart rate. Each patient performs exercise while being given real-time instructions by the instructor via the remote system, thereby guaranteeing safer exercise sessions than conventional methods. During exercise therapy sessions, the patient can video chat with the instructor through the system. Through this system, communication can be performed bidirectionally. The exercise sessions will be carried out on a 1-on-1 basis with the instructor for every patient. No serious complications have been previously reported in exercise therapies with respect to the appropriate exercise prescriptions [20-22]; thus, RCR is possibly safer than exercise performed by individual patients at their discretion. Moreover, educational instructions will be given 3 times weekly through e-learning methods. The e-learning content will include thorough information on the risks of disease, nutrition, lifestyle modification for disease prevention, guided exercise, and medication. Data on patients’ understanding of disease control will be collected. The e-learning videos will be made available for watching on tablets or other devices through the internet, using a proprietary app. The video content to be played will be determined by the medical team in accordance with relevance to the risks and diseases pertaining to that particular patient. Finally, to assess the understanding of the content, the patient will be asked to take a mini-test on a tablet. The mini-test is prepared for the content of each e-learning educational material. Subsequent educational programs will be adjusted on the basis of the results obtained. If issues are noted in monitoring devices or transmission issues, we shall stop the exercise session from a safety point of view and make an adjustment in the remote devices to resume subsequent exercise sessions. The clinical study will be conducted strictly in accordance with ethical guidelines and the latest revisions to the Declaration of Helsinki. This clinical trial is registered with the University Hospital Medical Information Network—Clinical Trials Registry (UMIN–CTR; UMIN000042942). Additionally, the protocol has been approved by the institutional review board of Tokyo University Hospital (2020305NI).

### Setting of Endpoints

The primary endpoint is the peak oxygen uptake 2-3 months from the start of exercise or 6-min walk test. The secondary endpoints are as follows: indices of cardiopulmonary exercise testing, occurrence rates of clinical events (all-cause deaths, cardiovascular deaths, and cardiovascular hospitalizations), N-terminal fragment of pro–B-type natriuretic peptide (NT-pro BNP) or BNP, health-related quality of life (QOL) scores, daily activity amounts, and questionnaire results. For both groups, various data items will be collected during regular checkups at and 2-3 months after hospital discharge, and improvements in exercise tolerance (peak oxygen intake) will be compared with the occurrence rates of clinical events.

### Data Collection

We will also collect the following data upon admission: (1) basic information such as facility name, record date, indications for cardiac rehabilitation, hospitalization date, birthdate, sex, height, weight, and social factors (ie, living alone, living with someone, and institutionalization); (2) patient background, including history of hospitalization for heart failure, underlying heart diseases (ischemic heart disease, heart failure, aortic and peripheral artery disease, valvular disorder, and congenital heart disease), concurrent diseases/complications (hypertension, diabetes, atrial fibrillation, cerebral stroke, peripheral vascular disease, chronic kidney disease, anemia, chronic obstructive pulmonary disease, and smoking); (3) treatment history (before hospitalization), including percutaneous coronary intervention, coronary artery bypass grafting, pacemaker placement, and valve surgery; (4) discharge day; (5) vital data (blood pressure and pulse rate); (6) cardiac disease severity in accordance with NYHA classification; (7) clinical laboratory data, including lymphocyte count, hemoglobin, creatinine, sodium, albumin, total bilirubin, uric acid, and BNP or NT-Pro BNP; (8) imaging data, such as electrocardiography, chest radiography, and echocardiography (including left ventricular end–diastolic diameter, left ventricular end–systolic diameter, left ventricular ejection fraction, interventricular septum width, left ventricular posterior wall width, valve lesions, transmitral flow pattern, mitral annular ring early diastole wave, left atrial volume index, and tricuspid regurgitation maximum blood velocity); (9) 6-min walk test and cardiopulmonary exercise testing; (10) questionnaires, health-related QOL scores, and sarcopenia scores; and (11) prescribed drugs. We will collect the following items 2-3 months after discharge (at an outpatient visit): (1) vital data; (2) NYHA classification; (3) clinical laboratory data; (4) imaging data; (5) 6-min walk test and cardiopulmonary exercise testing; (5) questionnaires, health-related QOL scores, and sarcopenia scores; and (6) prescribed drugs. We will also collect the following items 1 year after discharge (at an outpatient visit): all-cause deaths, cardiovascular-related deaths, and hospitalization due to cardiovascular disease (except for planned hospitalization).

### Data Analysis

The analysis will be performed as follows: data on the primary and secondary endpoints of the RCR and control groups will be expressed as mean (SD) or median (quadrant) values. JMP software (SAS Institute) will be used for statistical analysis. Continuous variables will be analyzed using a 2-tailed independent samples *t* test and the Mann–Whitney *U* test. For categorical variables, a chi-square test will be conducted. The RCR group will be compared with the historical control group at the primary and secondary endpoints. The sample size was calculated on the basis of the following estimation. The sample size has been estimated from the data of peak oxygen consumption. A difference in peak oxygen consumption, which is considered clinically relevant, will be in accordance with that reported previously [23,24]. Setting the 2-sided significance level at 5% and power at 80%, a sample size of 67 subjects per group will be required, after allowing for a 40% drop-out rate. Considering these issues, 3 cases are planned to be assigned to each institution (total 75 cases). The level of statistical significance will be set at *P*<.05.

## Results

This study was funded in December 2020 and received ethical approval in January 2021, and recruitment began in January 2021. In total, 59 patients have been recruited in the study by March 2021.

## Discussion

### Aim of This Trial

This trial aims to investigate the efficacy and safety of RCR in the recovery phase for patients with cardiovascular diseases. This RCR protocol includes 2 parts: (1) aerobic training using an ergometer, which will be installed in the patients’ homes and (2) patient education using an e-learning system. Safety during patients’ exercise will be ensured by monitoring multifaceted parameters such as blood pressure and heart rate, electrocardiography, and observation of patients during exercise through video chats. The e-learning system will promote an increased understanding of cardiovascular diseases.

### Strengths and Limitations

This trial is not randomized and observational; thus, background factors may not be accurately aligned with data on the control group, which will be based on clinical records. Results of exercise capacity is considered the primary outcome; however, the evaluation will be based on the 6-min walk test or a cardiopulmonary exercise test because the cardiopulmonary exercise test cannot be performed at all institutions, and some institutions can only measure exercise capacity by evaluating the 6-min walk test. This inconsistency in the evaluation of exercise capacity might decrease the statistical power of this study. Indeed, the compatibility of data between the cardiopulmonary exercise test and 6-min walk test has not been verified [25]. Laboratory data are measured at each facility, using the method prescribed at each facility, possibly resulting in differences in the laboratory data between each facility. Conversely, increasing the versatility of introducing RCR in this study might facilitate the application of the RCR protocol at various facilities.

### Potential Implications of This Trial

If this trial successfully confirms the efficacy of RCR, it will provide a valid alternative for patients who cannot participate in group-based outpatient rehabilitation programs because of various reasons. Moreover, the program may be applicable to diseases other than those explored in this study. The development of this modality may help overcome the requirement of recruiting patients in group-based OCR programs. Although this study focused only on cases in the recovery phase, patients in the maintenance phase may enroll under similar regimens. Furthermore, constructing a home rehabilitation environment monitored by a specialist will motivate the patient to manage his/her lifestyle habits and continue exercising. By constructing systems, teaching materials, and software applications for such RCR programs, we will be able to control not only disease prophylaxis/treatment but also lifestyle habits, including diet, sleep, and exercise. Hence, such systems will be utilized in diverse fields ranging from medicine, disease control, and health augmentation. The RCR protocol may provide remote intervention platforms for various health care professionals, including nutritionists, pharmacists, and exercise instructors, and apply to rehabilitation in other diseases (eg, cancer and cerebral infarction).

## Abbreviations

AI: artificial intelligence
AT: anaerobic threshold
BNP: B-type natriuretic peptide
NYHA: New York Heart Association
OCR: outpatient cardiac rehabilitation
QOL: quality of life
RCR: remote cardiac rehabilitation

## References

[1] Sandesara PB, Lambert CT, Gordon NF, Fletcher GF, Franklin BA, Wenger NK, Sperling L (2015). Cardiac rehabilitation and risk reduction: time to "rebrand and reinvigorate". J Am Coll Cardiol.

[2] Belardinelli R, Georgiou D, Cianci G, Purcaro A (1999). Randomized, controlled trial of long-term moderate exercise training in chronic heart failure: effects on functional capacity, quality of life, and clinical outcome. Circulation.

[3] Taylor RS, Brown A, Ebrahim S, Jolliffe J, Noorani H, Rees K, Skidmore B, Stone JA, Thompson DR, Oldridge N (2004). Exercise-based rehabilitation for patients with coronary heart disease: systematic review and meta-analysis of randomized controlled trials. Am J Med.

[4] Belardinelli R, Paolini I, Cianci G, Piva R, Georgiou D, Purcaro A (2001). Exercise training intervention after coronary angioplasty: the ETICA trial. J Am Coll Cardiol.

[5] Dunlay SM, Witt BJ, Allison TG, Hayes SN, Weston SA, Koepsell E, Roger VL (2009). Barriers to participation in cardiac rehabilitation. Am Heart J.

[6] Daly J, Sindone AP, Thompson DR, Hancock K, Chang E, Davidson P (2002). Barriers to participation in and adherence to cardiac rehabilitation programs: a critical literature review. Prog Cardiovasc Nurs.

[7] Grace SL, Midence L, Oh P, Brister S, Chessex C, Stewart DE, Arthur HM (2016). Cardiac Rehabilitation Program Adherence and Functional Capacity Among Women: A Randomized Controlled Trial. Mayo Clin Proc.

[8] McCartan F, Bowers N, Turner J, Mandalia M, Kalnad N, Bishop-Bailey A, Fu J, Clifford P (2017). Introduction of a novel service model to improve uptake and adherence with cardiac rehabilitation within Buckinghamshire Healthcare NHS Trust. BMC Cardiovasc Disord.

[9] Santiago de Araújo Pio C, Marzolini S, Pakosh M, Grace SL (2017). Effect of Cardiac Rehabilitation Dose on Mortality and Morbidity: A Systematic Review and Meta-regression Analysis. Mayo Clin Proc.

[10] Moulson N, Bewick D, Selway T, Harris J, Suskin N, Oh P, Coutinho T, Singh G, Chow C, Clarke B, Cowan S, Fordyce CB, Fournier A, Gin K, Gupta A, Hardiman S, Jackson S, Lamarche Y, Lau B, Légaré JF, Leong-Poi H, Mansour S, Marelli A, Quraishi AUR, Roifman I, Ruel M, Sapp J, Small G, Turgeon R, Wood DA, Zieroth S, Virani S, Krahn AD (2020). Cardiac Rehabilitation During the COVID-19 Era: Guidance on Implementing Virtual Care. Can J Cardiol.

[11] Ogura A, Izawa KP, Tawa H, Kureha F, Wada M, Harada N, Ikeda Y, Kimura K, Kondo N, Kanai M, Kubo I, Yoshikawa R, Matsuda Y (2021). Impact of the COVID-19 pandemic on phase 2 cardiac rehabilitation patients in Japan. Heart Vessels.

[12] Besnier F, Gayda M, Nigam A, Juneau M, Bherer L (2020). Cardiac Rehabilitation During Quarantine in COVID-19 Pandemic: Challenges for Center-Based Programs. Arch Phys Med Rehabil.

[13] Wakefield B, Drwal K, Scherubel M, Klobucar T, Johnson S, Kaboli P (2014). Feasibility and effectiveness of remote, telephone-based delivery of cardiac rehabilitation. Telemed J E Health.

[14] Su JJ, Yu DSF (2019). Effectiveness of eHealth cardiac rehabilitation on health outcomes of coronary heart disease patients: a randomized controlled trial protocol. BMC Cardiovasc Disord.

[15] Kikuchi A, Taniguchi T, Nakamoto K, Sera F, Ohtani T, Yamada T, Sakata Y (2021). Feasibility of home-based cardiac rehabilitation using an integrated telerehabilitation platform in elderly patients with heart failure: A pilot study. J Cardiol.

[16] Jolly K, Taylor RS, Lip GYH, Stevens A (2006). Home-based cardiac rehabilitation compared with centre-based rehabilitation and usual care: a systematic review and meta-analysis. Int J Cardiol.

[17] Blair J, Corrigall H, Angus NJ, Thompson DR, Leslie S (2011). Home versus hospital-based cardiac rehabilitation: a systematic review. Rural Remote Health.

[18] JCS Joint Working Group (2014). Guidelines for rehabilitation in patients with cardiovascular disease (JCS 2012). Circ J.

[19] Izawa H, Yoshida T, Ikegame T, Izawa KP, Ito Y, Okamura H, Osada N, Kinugawa S, Kubozono T, Kono Y, Kobayashi K, Nishigaki K, Higo T, Hirashiki A, Miyazawa Y, Morio Y, Yanase M, Yamada S, Ikeda H, Momomura S, Kihara Y, Yamamoto K, Goto Y, Makita S, Japanese Association of Cardiac Rehabilitation Standard Cardiac Rehabilitation Program Planning Committee (2019). Standard Cardiac Rehabilitation Program for Heart Failure. Circ J.

[20] Franklin BA, Bonzheim K, Gordon S, Timmis GC (1998). Safety of medically supervised outpatient cardiac rehabilitation exercise therapy: a 16-year follow-up. Chest.

[21] Scheinowitz M, Harpaz D (2005). Safety of cardiac rehabilitation in a medically supervised, community-based program. Cardiology.

[22] Bhat AG, Farah M, Szalai H, Lagu T, Lindenauer PK, Visintainer P, Pack QR (2021). Evaluation of the American Association of Cardiovascular and Pulmonary Rehabilitation Exercise Risk Stratification Classification Tool Without Exercise Testing. J Cardiopulm Rehabil Prev.

[23] Salvetti XM, Oliveira JA, Servantes DM, Vincenzo de Paola AA (2008). How much do the benefits cost? Effects of a home-based training programme on cardiovascular fitness, quality of life, programme cost and adherence for patients with coronary disease. Clin Rehabil.

[24] Servantes DM, Pelcerman A, Salvetti XM, Salles AF, de Albuquerque PF, de Salles FCA, Lopes C, de Mello MT, Almeida DR, Filho JAO (2012). Effects of home-based exercise training for patients with chronic heart failure and sleep apnoea: a randomized comparison of two different programmes. Clin Rehabil.

[25] Guazzi M, Dickstein K, Vicenzi M, Arena R (2009). Six-minute walk test and cardiopulmonary exercise testing in patients with chronic heart failure: a comparative analysis on clinical and prognostic insights. Circ Heart Fail.

